# Current Challenges in Prostate Cancer Management and the Rationale behind Targeted Focal Therapy

**DOI:** 10.1155/2012/862639

**Published:** 2012-05-10

**Authors:** Al B. Barqawi, Kevin J. Krughoff, Khadijah Eid

**Affiliations:** Division of Urology, University of Colorado Denver, Aurora, CO 80045, USA

## Abstract

Among men, prostate cancer has a high prevalence, with relatively lower cancer-specific mortality risk compared to lung and colon cancer. Prostate-specific antigen (PSA) screening has increased prostate cancer awareness since its implementation as a screening tool almost 25 years ago, but, due to the largely indolent course of this disease and the unspecific nature of the PSA test, increased incidence has largely been associated with cancers that would not go on to cause death (clinically insignificant), leading to an overdiagnosis challenge and an ensuing overtreatment consequences. The overtreatment problem is exacerbated by the high risk of side effects that current treatment techniques have, putting patients' quality of life at risk with little or no survival benefit. The goals of this paper are to evaluate the rise, prevalence, and impact of the overdiagnosis and ensuing overtreatment problems, as well as highlight potential solutions. In this effort, a review of major epidemiological and screening studies, cancer statistics from the advent of prostate-specific antigen screening to the present, and reports on patient concerns and treatment outcomes was conducted to present the dominant factors that underlie current challenges in prostate cancer treatment and illuminate potential solutions.

## 1. Background

Accounting for 29% of all cancers in men, prostate cancer is the most common cancer among men behind nonmelanoma skin cancer and is the second highest cause of cancer death among men of all races [1, 2]. Over 2 million men currently alive in the United States have had prostate cancer, and it is estimated that 16.48% of men will be diagnosed with prostate cancer at some point during their lives [3]. Estimates of newly diagnosed prostate cancer cases hover near 240,000 for 2011 [4].

However prevalent, the incidence and mortality of prostate cancer present very differently. It is estimated that 1 in 6 men will be diagnosed with prostate cancer but only 1 in 36 are expected to die because of it [5]. This may be because it is predominantly diagnosed in more senior adults, and, with a generally favorable outlook, men usually die before any symptoms appear [1, 5]. To be sure, there are tens of thousands of individuals who suffer the symptoms of aggressive prostatic cancer, but, in terms of the larger picture of prostate cancer, these men are well in the minority. The question remains, why, for such a largely symptomless condition, do so many incidental or nonmortal cancers get diagnosed, and what does a diagnosis of cancer mean at this clinically insignificant stage? The purpose of this paper is to understand the trends that made such a predominantly hidden cancer become so noticeable, highlight the burden that this now markedly prevalent cancer places on healthcare, and illuminate current developments that may hold promise for easing that burden.

## 2. PSA Test Increases Incidence

The primary reason for such a high rate of diagnosis for so often a symptomless condition is most likely the result of prostate-specific antigen (PSA) screening practices which came about in the late 1980s following studies which seemed to demonstrate the value of PSA as a biomarker for prostate cancer [6–8]. The 1987 study by Stamey and colleagues was perhaps the most dominant one due to its citation prevalence in Medline [9, 10]. In 2004, however, Stamey and colleagues maintained that PSA was only an accurate reflection of prostate cancer circa 1985 and that it was only demonstrated a relation to benign prostate hyperplasia throughout the five years preceding their newer study [11]. Moreover, Thompson and colleagues demonstrated in 2005 that there was no single PSA cutoff that could yield both high sensitivity and specificity [12].

**Figure 1:**
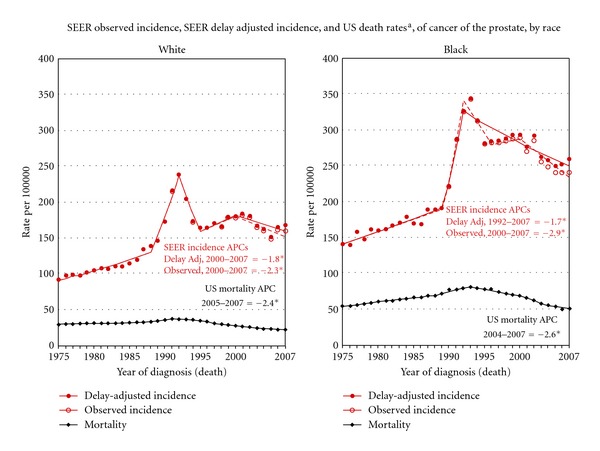
Change in prostate cancer incidence and mortality from 1975 to 2007 documented by the National Cancer Institute. ^a^rates are age adjusted to the 2000 US Std Population (19 age groups—Census P25-1103). Regression lines and APCs are calculated using the Joinpoint Regression Program Version 3.5, April 2011, National Cancer Institute. The APC is the annual percent change for the regression line segments. The APC shown on the graph is for the most recent trend. The APC is significantly different from zero (*P* < 0.05).

Nevertheless, early studies of PSA testing exhilarated the scientific community by offering the prospect of early detection of prostate cancer, a disease so often diagnosed late in its development due to its often symptomless progression [13, 14]. The use of PSA screening increased rapidly in the United States after 1987, resulting in a dramatic change in annual prostate cancer incidence and, in turn, a sharp increase in prostate cancer treatment both in the United States and abroad [15–17]. But while more cancers were being found and treated, the prostate cancer specific mortality rate only modestly decreased until 1993, after which little change was seen [15, 18].  Figure 1 from the National Cancer Institute shows this rising incidence of prostate cancer in contrast to the relatively unchanged mortality rate from 1975 to 2007. The disparity continues today, where the number of newly discovered prostate cancers is over seven times the number of prostate cancer related deaths [2].

## 3. PSA Test Creates Stage Migration

What may account for this is the fact that, while helping to discover mortal cancers, PSA testing also often led to the discovery of nonmortal cancers, or those which would never have been given notice in the absence of screening [19]. Given that 20–50% of asymptomatic men are found to harbor prostate cancer upon autopsy, it follows that the PSA test, with only a 24.1% positive predictive value, leads to a much greater detection of cancers, both mortal and nonmortal [20–23]. It is also possible that widespread PSA testing and treatment may have slowly weeded out the more dangerous prostate cancers from the population. Whether from increased testing, increased treatment of dangerous cancers, or some combination of the two, more cancers were being found at lower stages from 1986 to 1993, with tumors often being low grade, clinically localized, and/or organ confined. From 1993 to 2003, there was a 75% reduction in the proportion of metastatic diagnoses for prostate cancer [24]. The link between PSA testing and stage migration was documented in Austria during a large-scale PSA testing study and again later in the United States [24–26]. This seems to indicate that as PSA testing continues, prostate cancer will also continue to be diagnosed at clinically insignificant stages.

## 4. Overdiagnosis Ensues

Given the propensity of PSA testing to detect cancers both mortal and nonmortal, overdiagnosis was a probable outcome. Overdiagnosis due to PSA testing has been documented extensively through epidemiological studies and computerized models, at rates which range from 29% in specific regions to an estimated 80% should all men in the United States be screened [7, 27, 28]. Progress has been made in investigating different biomarkers and variations of PSA testing for early detection of mortal prostate cancer, but, despite its flaws, the PSA test still remains the best screening tool currently available, suggesting continued overdiagnosis [29–31].

## 5. Uncertainty Leads to Treatment

The corollary of the overdiagnosis problem is an overtreatment problem. While active surveillance (AS) might seem the best course of action for many due to the relatively low mortality rate and exceedingly high 15-year survival rates of prostate cancer, working against that is a lack of consensus on what the inclusion criteria should be for AS, what the optimal follow-up schedule should be, or even how to best define progression [32]. For instance, the Epstein criteria is one common method of establishing whether or not a cancer is clinically insignificant, and this relies on a third or less of biopsy cores being positive, 50% or less involvement of any 1 core, and a PSA density of less than 0.15 ng/mL. However, the D'Amico criteria, also widely used, calls for a Gleason score of six or less, a PSA of less than 10 ng/mL, and a T1 clinical stage. Studies have shown highly favorable results for certain criteria, like the 100% 10-year prostate cancer-specific survival rate documented by researchers who took patients off AS based on PSA doubling time [33]. Other studies suggest that PSA kinetics are not reliable for AS inclusion/exclusion criteria [34]. In yet another study, researchers found that prostate specimens fitting six different inclusion criteria for clinically insignificant disease would have been misclassified 14–27% of the time based on Gleason 8 findings [35]. Research continues to refine AS criteria, but a clear understanding of how to define clinically insignificant disease has not been reached.

The lack of consensus and the thought of harboring a cancer with an unpredictable progression leads to feelings of uncertainty in the patient, in turn arousing high levels of emotional distress, anxiety, and depression [36]. While support services can assist in ameliorating the psychological distress, men with prostate cancer tend to avoid disclosure and are unlikely to utilize health and psychological support services [37]. At the same time, doctors tend to underestimate the psychological morbidity of men with prostate cancer, leading to a lack of provider referral [38].

With a lack of social support, motivation to seek it out, or provider referrals to address the psychological discomfort associated with prostate cancer, most newly diagnosed men suffer the full psychological burden of living with an unpredictable cancer. This proves too much to bear, as rather than learning to live with what is most likely a nonmortal cancer, men elect various courses of treatment to escape the mental anguish of uncertainty. In a study of the reasons for undergoing various treatment types, Gwede et al. found that 44% of men chose radical prostatectomy primarily because they believed it to be their best chance to be cured [39].

Denberg et al. found that a group of men underwent surgery due to the belief that it was the most certain, expeditious, and tangible option and that, even though it might reveal that a tumor escaped the prostate, it would at least eliminate some uncertainty. These same men found no other option appealing because they dealt with acting on a hidden and unseen cancerous organ. Even those who did not choose surgery in the Denberg study were motivated by uncertainty, in their case, they were trying to avoid the uncertainties associated with surgery. It should also be noted that half of their patient sample avoided seeking second opinions due to delay, prolonged uncertainty, and feelings of increased anxiety [40]. Similar findings were reported in England, Scotland, and Whales, where a study of 50 men with early-stage prostate cancer found reasons for prostatectomy ranging from frustration with the lack of concrete information and consensus over what to do, to the explicit desire to fix the problem [41].

The uncertainty and anxiety of having prostate cancer are certainly a formidable driver for treatment instead of AS, and researchers have documented it as a valuable predictor of treatment receipt [42, 43]. Watchful waiting (often synonymous with AS) is often used in other countries but rarely in the United States, especially for younger men with early-stage prostate cancer for whom treatment is often advocated [44]. When briefed with specific cancer statistics and information on the side effects of treatment, most patients place little weight on side effects when there is even a chance of prolonged survival [45]. Mazur and Hickam showed that even when attempting to bias patients against surgical therapy by explicitly naming surgical complications and presenting rates of those complications higher than what was typically in the literature, most patients still preferred surgical treatment over AS for localized prostate cancer [46]. Research shows that, on the whole, only 18.5% forgo active treatment for watchful waiting, all in the face of a cancer that is lethal in only 1 in 32 cases [47]. 

## 6. Prevalence of Overtreatment

The amount of treatment received is certainly disproportional, and studies clearly indicate that a substantial proportion of treatments do not go on to prevent death from prostate cancer. The European Randomized Study for Prostate Cancer (ERSPC) reported, for instance, that 1410 men needed to be screened and 48 treated to prevent 1 cancer death [48]. Results from the Randomized Scandinavian Prostate Cancer Group Study show that an estimated 15 patients needed to be treated to avert one death at 15 years and that, for adjuvant radiation therapy, the number of patients needed to be treated to avert one death at 12.6 years was 9.1 [49]. Perhaps the most favorable results were found in Quebec, where out of an estimated 100 men with screen-detectable prostate cancer, an average of 16 could have their lives extended by surgery (should those men be found by way of extensive screening efforts) [50]. However, the most recent data comes from the Prostate Cancer Intervention versus Observation Trial (PIVOT), which reports that, after 12 years of followup, overall prostate cancer mortality was only 3% lower for men having radical prostatectomy. In fact, men with low-risk prostate cancer were actually shown to have a 2.4% better survival rate with watchful waiting than with surgery. PIVOT reports that, even when looking exclusively at cases of intermediate risk, radical prostatectomy still only achieves a 4.8% better survival rate [51].

The statistics from these studies also do not take into account the copious amounts of unnecessary biopsies that would have to be performed to find these cancers in the first place, as in order to detect even 83.4% of cancer cases by PSA testing, a calculated 61.1% of men without cancer would need to be subjected to prostate biopsy, a procedure that is in itself not without consequence to quality of life [12].

In an effort to quantify the amount of overtreatment stemming from prostate cancer, Welch and Albertsen used Surveillance Epidemiology and End Results (SEER) data from the National Cancer Institute and statistics from the US Census to estimate that from 1986 to 2005, 1,004,800 of an additional 1,305,600 overdiagnosed cancers received treatment, with 571,000 excess prostate-cancer-related surgeries, and 477,400 excess prostate-cancer-related radiation treatments [10].

## 7. Side Effects of Treatment

Excess treatment brings excess side effects, and, in the case of prostate cancer, they are not uncommon. Overtreated patients run several risks, especially when it comes to radical prostatectomy and/or radiation therapy, the most dominant treatment options. The US Preventative Services Task Forces reviewed the most common side effects of treatment from 1994 to 2002, bringing the problems associated with overtreatment to light. Long-term adverse effects of radical prostatectomy, for instance, were sexual dysfunction (20–70%) and urinary incontinence (15–50%). For electron beam radiation therapy, approximately 45% could expect erectile dysfunction, 2–16% urinary dysfunction, and 6–25% bowel dysfunction. For Androgen Deprivation Therapy (ADT), approximately half of patients who were sexually active beforehand were not sexually active afterward, 5–25% had breast swelling, and 50–60% had hot flashes along with other potential long-term complications like anemia and osteoporosis. For brachytherapy, a majority of men reported having distressing urinary symptoms, 21–36% reported decreased erectile function, 18% diarrhea, and 19% persistent rectal bleeding [52].

The primary concern of most men undergoing radical prostatectomy is preservation of potency, and, to that end, bilateral nerve sparing techniques in younger cohorts (median age 57) have yielded a potency rate as high as 86%, with more typical results hovering around 44–76% [53]. The second most dominant concern is urinary continence, but data on that is difficult to generalize given the changing definition of continence which various studies employ. When urinary continence is defined as not needing protection to keep outer garments dry, 93% of men followed for more than 18 months recovered continence, but, when using total urinary control as a benchmark, only 32% of men were continent at 24 months [53]. Among patients in the Rotterdam section of the ERSPC who underwent radical prostatectomy, as much as 80–90% reported erectile dysfunction and 39–49% reported urinary incontinence [54]. Studies analyzing even the most advanced minimally invasive prostatectomy techniques (robotic and/or laparoscopic) find continence ranging 68.0–94.7%, potency 33.3–65.3%, and progression-free survival 84.1–92.0% [55].

In more recent findings, these problems are still prevalent. In a comparison of 1938 men who received minimally invasive radical prostatectomy and 6899 men who received open retropubic radical prostatectomy, investigators found incontinence rates of 15.9 and 12.2 per 100 person-years, respectively. Sexual dysfunction was higher, however, with rates of 26.8 and 19.2 per 100 person-years, respectively [56]. Another large-scale study of 1,201 patients treated with surgery, brachytherapy, or EBRT found erectile dysfunction rates two years posttreatment of 57%, 31%, and 35%, respectively [57, 58].

## 8. Focal Therapy Offers a Solution

The aforementioned statistics indicate that the majority of these treatments will infer no survival benefit in the first place, so the amount of men who go on to suffer such side effects is certainly unwarranted. However, without an established and reliable way to distinguish mortal from nonmortal cancers and the overwhelming preference by both patients and providers to pursue treatment options in the face of such uncertainty, it seems treatment will continue to be the dominant option. Fortunately, focal therapy techniques developing since the 1990s are now showing promise as a method of treatment which is not associated with such arresting rates of side effects. These techniques avoid the costs that other techniques would require in order to reduce side effects to comparable levels while still being effective [59].

Clinical trials have demonstrated the feasibility of focal ablative methods using high-intensity focused ultrasound and cryosurgery [60], and focal techniques are expected to improve as imaging techniques allow for better pathological assessments [61–63]. Successful focal therapy demands stringent selection factors, and this requires an imaging modality which can accurately characterize the location, extent, and grade of a patient's prostate cancer [64]. In this effort, a brachytherapy template-guided transperineal saturation biopsy technique (3DMB) was described and tested by Crawford et al. on prostate autopsy specimens in 2005, which demonstrated both feasibility and increased accuracy over sextant biopsies [65]. In evaluating the potential of 3DMB in vivo, Barqawi et al. compared previous TRUS results of 215 patients to those obtained using 3DMB, finding new cancer foci in 82 patients and higher Gleason scores in 49 patients, demonstrating a potentially significant improvement in the way prostate cancer can be evaluated [66]. While long-term results have not been disseminated as of yet, we are currently anticipating the publication of 5-year follow-up data on patients treated with focal cryoablation in conjunction with 3DMB.

While promising results continue to be seen, there are still hurdles to overcome, such as the issue of gland stabilization during treatment, or how to work around large prostates when employing 3DMB [67]. In addition, wide variability in patient selection, disease characterization, and treatment protocols still exist. A preferred ablative energy for focal therapy has also not been conferred (cryosurgery, high-intensity focused ultrasound, vascular-targeted photodynamic therapy, brachytherapy, radiotherapy, or tomotherapy), yet it seems that one of the dominant concern for those investigating this approach has to do with standardizing follow-up protocols and creating reliable and meaningful outcomes measures to evaluate it, as the PSA measurements so often relied on to assess other forms of treatment are not only unreliable as mentioned, but also tend to take on different meaning when larger portions of the organ are left intact, as is the case with focal therapy [68, 69]. While biochemical disease-free status using American Society of Therapeutic Radiation Oncology or Phoenix criteria seems to be the dominant means of evaluating focal treatment, standardized algorithms for determining success would be of significant benefit to researchers pursuing long-term follow-up studies on focal therapy. Standardization should be a primary focus of future follow-up studies and will serve greatly in conferring the efficacy of different methods and identifying areas for improvement.

## 9. Conclusion

While procedural advances and screening efforts continue to report improvements, the PSA test is still the best screening tool currently available, and this suggests a continuing trend of overdiagnosis based on historical data. Early detection of prostate cancer is possible, but early discrimination is not, leading to a great deal of uncertainty as to whether or not a particular patient's prostate cancer will become aggressive. The psychological burden that comes with this uncertainty more often than not leads to treatment regardless of patients' understanding of high risks of side effects and low survival benefit rates. Whether or not improved screening or imaging techniques will be able to better distinguish nonmortal from mortal cancers remains to be seen, as well as what role that will play in regards to the psychological distress that comes with being diagnosed with prostate cancer.

With overwhelming evidence, the root of overtreatment and the unnecessary side effects that ensue lie in the psychological burden of dealing with uncertainty and with the lack of emotional support or the motivation to seek it out, the vast majority of newly diagnosed men undergo serious treatment efforts regardless of the potential for harmful side effects. Solutions to the overtreatment problem may come from enhanced screening and imaging efforts or the delivery and implementation of psychological care for those diagnosed with localized cancer. More likely, solutions will come from improvements in treatment methodology. Focal therapy appears to be a promising avenue in this regard as it is noninvasive, has fewer side effects, and remains more cost-effective than side-effect reducing advanced radiation and robotic techniques. Moreover, focal therapy does not exclude the possibility of more radical options and does not necessarily replace traditional techniques. Enhanced methods to evaluate focal therapy and standardized protocols to assess outcomes measures will help progress this emerging practice as improvements in imaging modalities help practitioners realize its assumed potential.
